# RET splice site variants in medullary thyroid carcinoma

**DOI:** 10.3389/fgene.2024.1377158

**Published:** 2024-03-19

**Authors:** Daryoush Saeed-Vafa, Kyriakos Chatzopoulos, Juan Hernandez-Prera, Pedro Cano, James J. Saller, Julie E. Hallanger Johnson, Bryan McIver, Theresa A. Boyle

**Affiliations:** H. Lee Moffitt Cancer Center, Tampa, FL, United States

**Keywords:** medullary thyroid carcinoma, RET, splice site variant, thyroid, endocrine

## Abstract

**Introduction:** Medullary thyroid carcinoma (MTC) is an aggressive cancer that is often caused by driver mutations in *RET*. Splice site variants (SSV) reflect changes in mRNA processing, which may alter protein function. *RET* SSVs have been described in thyroid tumors in general but have not been extensively studied in MTC.

**Methods:** The prevalence of *RET* SSVs was evaluated in 3,624 cases with next generation sequence reports, including 25 MTCs. Fisher exact analysis was performed to compare *RET* SSV frequency in cancers with/without a diagnosis of MTC.

**Results:** All 25 MTCs had at least one of the two most common *RET* SSVs versus 0.3% of 3,599 cancers with other diagnoses (*p* < 0.00001). The 11 cancers with non-MTC diagnoses that had the common *RET* SSVs were 4 neuroendocrine cancers, 4 non-small cell lung carcinomas, 2 non-MTC thyroid cancers, and 1 melanoma. All 25 MTCs analyzed had at least one of the two most common *RET* SSVs, including 4 with no identified mutational driver.

**Discussion:** The identification of *RET* SSVs in all MTCs, but rarely in other cancer types, demonstrates that these *RET* SSVs distinguish MTCs from other cancer types. Future studies are needed to investigate whether these *RET* SSVs play a pathogenic role in MTC.

## Introduction

Medullary thyroid carcinoma (MTC) is a rare but aggressive carcinoma that arises from the neuroendocrine parafollicular C cells of the thyroid (NCCN, 2022). MTC represents 1%–2% of all thyroid cancers in the United States—they can occur sporadically, 80%, or via the germline within the spectrum of multiple endocrine neoplasia 2 (MEN2) syndrome (NCCN, 2022; Thyroid, 2023). The 5-year relative survival for stage I and II MTC is approximately 95%, whereas the 5-year survival for stage IV is approximately 28% (Hundahl et al., 1998; Edge and Compton, 2010). *RET*, a 20-exon proto-oncogene, encodes a tyrosine kinase receptor involved in the control of cell differentiation and proliferation. *RET* alterations play a significant role in the development and progression of MTC (Chakravarty et al., 2017). Somatic *RET* mutations have been reported in 40%–65% of sporadic MTCs and germline *RET* gain-of-function alterations predispose individuals to MEN2 syndrome (Boichard et al., 2012; Ciampi et al., 2013; Verrienti et al., 2016; Ciampi et al., 2019; Verrienti et al., 2022).

Splice site variants (SSV) are genetic mutations that occur in the regions of the genome that control the way pre-messenger RNA is processed. Specifically, these variants occur in the sequences of nucleotides that direct the removal of introns (non-coding regions) and the joining of exons (coding regions) to form mature messenger RNA. Splice variants can lead to the production of abnormal or truncated proteins, with potentially altered functional properties, that may have a significant impact on cell biology and tumorigenesis (Chen et al., 2015).

The literature about splice variants in thyroid cancers is limited. Two functional isoforms, *RET51* and *RET9*, formed via alternative splicing near the C-terminus (3’ end) of RET have been found to play distinct roles in tumorigenesis and/or development (Richardson et al., 2012). Furthermore, both *RET51* and *RET9* functional isoforms have been identified in MTC; however, neither played a role in MTC tumorigenesis (Mule et al., 2021). Lorenzo et al. described three alternative splice variants in a MTC cell line (Lorenzo et al., 1995). These variants were described as lacking only exon 3 (*RET* 2/4), lacking exons 3 and 4 (*RET* 2/5), and lacking exons 3, 4, and 5 (*RET* 2/6). A study of papillary thyroid cancer (PTC) biopsies in 2001 identified different splice variants in the *RET* extracellular domain described as in-frame changes with preservation of the tyrosine kinase domain (*RET* 1/8, *RET* 2/8, *RET* 3/8) (Fluge et al., 2001). In this study, they also identified *RET* 3/8 in two MTC biopsies and *RET*2/8 in one MTC biopsy. In 2002, a study of germline (MEN2-related) and sporadic pheochromocytomas described the presence of the same 3 *RET* splice variants identified in the MTC cell line in pheochromocytomas (*RET* 2/4, *RET* 2/5 and *RET* 2/6) (Le Hir et al., 2002). In 2010, McIver et al. described how abnormal RNA processing may be common in thyroid neoplasms with a possible pathogenetic role (McIver et al., 2000). However, to the best of our knowledge, *RET* splice variants have not been extensively studied in clinical samples diagnosed with MTC.

In this study, we used retrospective clinical next-generation sequencing (NGS) results to identify *RET* splice variants and aimed to explore their frequency and potential significance for MTC diagnosis.

## Materials and methods

An IRB-approved retrospective review of our institutional database of NGS results from 2018 to 2022 was performed to identify all sequenced cases with a pathological diagnosis of MTC. The database includes results from testing lung, colorectal, melanoma, thyroid, neuroendocrine, pancreatic, soft tissue, prostate, bladder, kidney, head and neck, hepatocellular, gastroesophageal, endometrial, ovarian, cervical, and other various cancers. We then reviewed these cases and recorded all molecular variants, including *RET* splice variants. We also queried the same database to identify all cases, irrespective of tumor histology, with *RET* splice variants. NGS was performed with the Illumina TruSight Tumor 170 (Illumina Inc., San Diego, CA) platform, a hybrid-capture 170-gene panel designed to identify clinically important small variants and copy number variants by DNA-based testing and splice variants and fusions by simultaneous RNA-based testing of tissue from solid tumors (Boyle et al., 2021). For a case to be considered positive for a *RET* splice variant, it needed to have passed RNA quality control (QC) metrics (including greater than 1,000,000 total uniquely mapped reads) with a “true” *RET* splice variant identified by the Illumina TruSight Tumor 170 pipeline and/or have ≥10 splice variants reads identified via sequence review in Integrative Genomics Viewer (IGV, Broad Institute) software (Robinson et al., 2011). Cases which failed these parameters or had inadequate tumor cellularity (<20%) to properly assess for *RET* splice variants were excluded. Only cases that passed QC metrics were included in this study. When applicable, results from a targeted seven gene thyroid cancer panel (Invitae Genetics, San Francisco, CA) were used for *RET* germline analysis. We performed a Student’s t-test to compare the tumor size between primary MTCs with two specific *RET* SSV *versus* those with just one and Fisher Exact analysis to compare the frequency of *RET* splice variants in MTC *versus* non-MTC cases.

## Results

**FIGURE 1:**
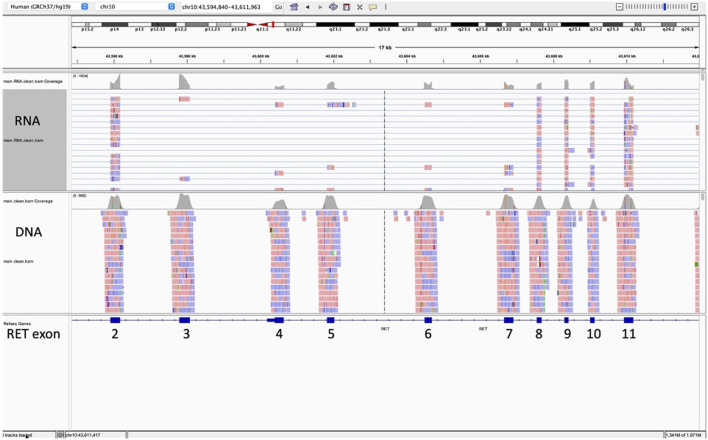
This Integrative Genomics Viewer (IGV) snapshot of sequence (horizontal lines) for RET exons 2 through 11 represents the results for a medullary thyroid carcinoma with RET splice variant 2/8. This splice variant splices the 3′ end of exon two amino acid 113 to the 5′ end of exon eight amino acid 508 with skipping of exons three through 7 (NM_20975.6:r.339_1522del). The splice variant is represented by the blue horizontal lines connecting sequence from exon 2 (forward is red; reverse is blue) directly to exon 8 with no sequence (no red or blue boxes) for exons three to 7.

**FIGURE 2:**
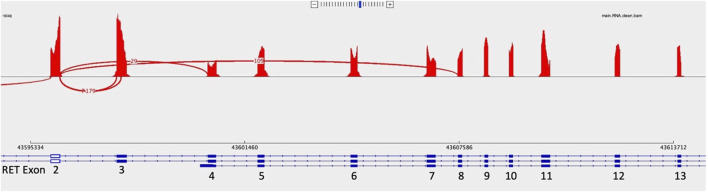
Sashimi plots represent a way to visualize splicing between exons. In this Sashimi plot from a medullary thyroid carcinoma case with a RET 2/8 splice variant, a curved red line represents sequence reads with alternative splicing from the end of exon two through exon 8, skipping exons 3 through 7.

Of the 3,624 cases with NGS results in our institutional database, 37 (1.0%) had a pathological diagnosis of MTC, either primary or metastatic. Of these 37 cases, 12 were excluded due to not meeting the inclusion requirements of this study (tumor cellularity >20% and at least 1,000,000 total unique RNA reads). Of the 25 remaining MTC cases, all (100%) had at least one *RET* splice variant, with most having multiple. All 25 had at least one of the following two in-frame *RET* splice variants, t (10; 10) (q11.2; q11.2) (chr10:g.43596172chr10:g.43607546), which we will label *RET* 2/8 to be consistent with the *RET* splice variant labeling by Le Hir et al. (Le Hir et al., 2002), or t (10; 10) (q11.2; q11.2) (chr10:g.43596172chr10:g.43600398), which we will label *RET* 2/4. *RET* 2/8 splices the 3′ end of exon two amino acid 113 to the 5′ end of exon eight amino acid 508 resulting in the skipping of exons three through 7 (NM_20975.6:r.339_1522del, Figures 1, 2). *RET* 2/4 splices the 3′ end of exon two amino acid 113 to the 5’ end of exon four amino acid 209 resulting in the skipping of exon 3 (NM_20975.6:r.339_625del).

*RET* 2/8 was identified in 96% of the MTC cases (24/25; 96%) with supporting reads ranging from 10 to 318. We reviewed the sequence of the one negative case for *RET* 2/8 in IGV and identified four reads consistent with *RET* 2/8 which was below our reportable threshold of 10 reads to definitively call the case positive for *RET* 2/8. *RET* 2/4 was identified in 76% (19/25; 76%) of MTC cases with supporting reads ranging from 10 to 123. Neither *RET* 2/8 nor *RET* 2/4 impacted expression of the tyrosine kinase domain, which spans amino acid 724 to amino acid 1005, exons 12 to 19 (Gabreski et al., 2016; Chakravarty et al., 2017; Paratala et al., 2018). Both *RET* 2/8 and *RET* 2/4 were identified in 72% of the MTC cases (18/25; 72%). Other splice variants were identified, but at lower frequency.

Of the 25 MTC cases with *RET* splice variants, 16 (64%) had a concurrent *RET* mutation with p.M918T as the most prevalent (9/16; 56%) mutation. Of the 16 patients with concurrent *RET* splice variants and mutations, 12 had a follow-up *RET* germline test. A germline origin of the mutation was identified in 2 (16.7%) of the 12 resulting in diagnoses of MEN type 2A. The *RET* germline tests for the other 10 patients were negative, consistent with a somatic origin for the cancers. Of the 9 MTC splice variant positive cases without a concurrent *RET* mutation, 5 (56%) had *HRAS* mutations, with p. Q61R (3/5; 60%) as the most prevalent specific mutation. There were 4 (4/25; 16%) *RET* splice variant positive MTCs with no significant driver mutation identified. Of the 5 cases without *RET* 2/4, 4 (4/5, 80%) had *RET* mutations and 1 (1/5, 20%) had a *HRAS* mutation without a concurrent *RET* mutation (Table 1).

**TABLE 1:** Medullary Thyroid Carcinomas evaluated for *RET* splice site variants.

Patient #	Sex (M/F)	Pathologic diagnosis	RET 2/8 (Y/N)	RET 2/4 (Y/N)	RET mutations	RET mutation germline/Somatic	Other significant mutations
1	F	primary MTC	Y	Y	p.C634W	Somatic	None
2	F	primary MTC	Y	N	None	N/A	HRAS p.Q61R
3	F	primary MTC	Y	Y	None	N/A	None
4	F	primary MTC	Y	Y	p.C634R	Somatic	None
5	F	primary MTC	Y	N	p.M918T	Somatic	None
6	F	primary MTC	Y	Y	None	N/A	None
7	F	primary MTC	Y	Y	p.M918T	Not performed	None
8	M	primary MTC	Y	Y	p.C630R	Somatic	None
9	M	primary MTC	Y	Y	None	N/A	None
10	F	primary MTC	Y	N	p.M918T	Somatic	None
11	F	primary MTC	Y	Y	p.C618S	Germline	None
12	F	Metastatic MTC to LN	Y	Y	None	N/A	HRAS p.Q61R
13	F	Metastatic MTC to LN	Y	Y	p.M918T, p.V804L, p.A883V	Not performed	None
14	F	Metastatic MTC to LN	Y	Y	p.M918T	Not performed	None
15	F	Metastatic MTC to LN	Y	Y	p.M918T	Not performed	None
16	M	Metastatic MTC to LN	Y	N	p.D898_E901del	Somatic	None
17	F	Metastatic MTC to LN	Y	N	p.C634R	Germline	None
18	M	Metastatic MTC to LN	Y	Y	p.M918T	Somatic	None
19	M	Metastatic MTC to LN	Y	Y	None	N/A	HRAS p.K117N
20	F	Metastatic MTC to LN	Y	Y	None	N/A	HRAS p.Q61R
21	M	Metastatic MTC to Liver	Y	Y	None	N/A	HRAS p.G13R
22	F	Metastatic MTC to left neck mass	Y	Y	None	N/A	None
23	F	Metastatic MTC to Bone	Y	Y	p.M918T	Somatic	None
24	F	MTC involving soft tissue	Y	N	p.M918T	Somatic	None
25	F	Metastatic MTC to Breast	N	Y	p.D631_E632del	Somatic	IDH1 p.R132C

MTC , medullary thyroid carcinoma; LN , lymph node.

RET, 2/8 = t(10; 10) (q11.2; q11.2) (chr10:g.43596172:chr10:g.43607546).

RET, 2/4 = t(10; 10) (q11.2; q11.2) (chr10:g.43596172:chr10:g.43600398).

Nine of the 11 primary MTCs had associated pathological data. The size of these nine primary MTCs tumors ranged from one to 3.8 cm with an average size of 2.1 cm and a median of 1.6 cm. Of the nine primary MTCs, six had both *RET* SSV 2/8 and *RET* SSV 2/4, while three had only *RET* SSV 2/8. The three primary MTCs with only *RET* SSV 2/8 measured 1, 1.2, and 3.8 cm with an average of 2 cm and a median of 1.2 cm *versus* a range of 1.2–3.7 cm with an average of 2.1 cm and median of 1.7 cm for the six primary MTCs with both *RET* SSV 2/8 and *RET* SSV 2/4. There was no statistically significant difference in tumor size between the two groups (t (9) = 0.16, *p* = 0.88; Table 2).

**TABLE 2:** Pathological data associated with *RET* splice site variant positive medullary thyroid carcinomas.

Patient #	Sex (M/F)	Pathologic diagnosis	RET 2/8 (Y/N)	RET 2/4 (Y/N)	Tumor size (cm)	Lymphatic invasion (Y/N)	Vascular invasion (Y/N)	Perineural invasion (Y/N)	Extra-thyroidal extension (Y/N)	AJCC stage
1	F	primary MTC	Y	Y	1.2	N	N	N	N	I
2	F	primary MTC	Y	N	3.8	Y	N	N	N	III
3	F	primary MTC	Y	Y	NR	NR	NR	NR	NR	NR
4	F	primary MTC	Y	Y	NR	NR	NR	NR	NR	NR
5	F	primary MTC	Y	N	1.0	N	N	N	Y	I
6	F	primary MTC	Y	Y	1.7	N	N	NR	N	NR
7	F	primary MTC	Y	Y	1.6	N	N	NR	N	III
8	M	primary MTC	Y	Y	1.6	Y	Y	NR	N	NR
9	M	primary MTC	Y	Y	3.7	Y	N	NR	Y	NR
10	F	primary MTC	Y	N	1.2	NR	N	NR	NR	NR
11	F	primary MTC	Y	Y	3	Y	N	N	Y	IVA
12	F	Metastatic MTC to LN	Y	Y	NR	NR	NR	NR	NR	III
13	F	Metastatic MTC to LN	Y	Y	NR	NR	NR	NR	NR	III
14	F	Metastatic MTC to LN	Y	Y	NR	NR	NR	NR	NR	III
15	F	Metastatic MTC to LN	Y	Y	NR	NR	NR	NR	NR	III
16	M	Metastatic MTC to LN	Y	N	NR	NR	NR	NR	NR	III
17	F	Metastatic MTC to LN	Y	N	NR	NR	NR	NR	NR	III
18	M	Metastatic MTC to LN	Y	Y	NR	NR	NR	NR	NR	III
19	M	Metastatic MTC to LN	Y	Y	NR	NR	NR	NR	NR	III
20	F	Metastatic MTC to LN	Y	Y	NR	NR	NR	NR	NR	III
21	M	Metastatic MTC to Liver	Y	Y	NR	NR	NR	NR	NR	IVC
22	F	Metastatic MTC to left neck mass	Y	Y	NR	NR	NR	NR	NR	IVC
23	F	Metastatic MTC to Bone	Y	Y	NR	NR	NR	NR	NR	IVC
24	F	MTC involving soft tissue	Y	N	NR	NR	NR	NR	NR	IVC
25	F	Metastatic MTC to Breast	N	Y	NR	NR	NR	NR	NR	IVC

MTC , medullary thyroid carcinoma; LN , lymph node; NR , not reported.

RET, 2/8 = t(10; 10) (q11.2; q11.2) (chr10:g.43596172:chr10:g.43607546).

RET, 2/4 = t(10; 10) (q11.2; q11.2) (chr10:g.43596172:chr10:g.43600398).

At least one *RET* splice variant was identified in 11 of 3,599 (0.3%) non-MTC cases. Of these 11 cases, four were neuroendocrine carcinomas (4.7%, 4/86 neuroendocrine carcinomas), four were non-small cell lung carcinomas (0.6%, 4/697 non-small cell lung carcinomas), two were non-MTC thyroid cancers (1.2%, 2/168 non-MTC thyroid cancers) (1 PTC (1.2%, 1/82 PTCs), and one not otherwise specified), and one was a melanoma (0.1%, 1/791 melanomas). Of these 11 non-MTC cancers with a *RET* splice variant, eight harbored *RET* 2/8, six had *RET* 2/4, and six had both splice variants. One case, a mixed neuroendocrine non-neuroendocrine neoplasm (MINEN), had a *RET* splice variant different than *RET* 2/8 and *RET* 2/4 (Table 3).

**TABLE 3:** Non-medullary thyroid carcinomas evaluated for *RET* splice site variants.

Patient #	Sex (M/F)	Pathologic diagnosis	RET 2/8 (Y/N)	RET 2/4 (Y/N)	RET mutations	Other significant mutations
1	F	Metastatic moderately differentiated neuroendocrine carcinoma (atypical carcinoid tumor)	Y	Y	None	HRAS p.Q61L
2	F	High-grade neuroendocrine carcinoma with features of small cell carcinoma	Y	Y	None	EFGR p.E746_A750del; TP53 p.N311_F328delinsHT; RB1 p.F684del
3	F	High-grade neuroendocrine carcinoma, with small cell features	Y	N	None	TP53 p.R158L; RB1 p.A22Gfs*9
4	M	Mixed neuroendocrine non-neuroendocrine neoplasm (MINEN) of the liver	N	N	None	KRAS p.Q61R; TP53 p.Y236*
5	M	Non-small cell lung cancer	Y	Y	None	BRAF p.G469V; TP53 p.P152Lfs*29
6	M	Metastatic lung adenocarcinoma to liver	Y	Y	None	TP53 p.V157F
7	F	Metastatic lung cancer to brain	Y	Y	None	KRAS p.G13C, TP53 p.R249T; TP53 p.R158H
8	M	Metastatic lung cancer to lymph node	Y	Y	None	TP53 p.Y236N; STK11 p.Y166*
9	M	Melanoma	U	U	None	BRAF p.L597R; TERT promoter c.-124C>T
10	F	Papillary thyroid cancer	Y	N	CCDC6-RET fusion	None
11	M	Thyroid cancer, not otherwise specified	U	U	None	TP53 p.K120R; PTEN p.Y27N

Y = yes; N = no; U = unable to identify specific location of RET, splice variant due to unavailability of file for detailed review in Integrated Genomic Viewer (IGV).

RET, 2/8 = t(10; 10) (q11.2; q11.2) (chr10:g.43596172:chr10:g.43607546).

RET, 2/4 = t(10; 10) (q11.2; q11.2) (chr10:g.43596172:chr10:g.43600398).

Overall, a total of 36 of the 3,624 (0.99%) cases were positive for a *RET* splice variant, of which 25 (69%) were MTC. All 25 MTC cases were *RET* splice variant positive *versus* 11 of 3,599 total non-MTC cases (100% *versus* 0.3%, *p* < 0.00001).

## Disscussion

Medullary thyroid carcinoma is a rare aggressive form of thyroid cancer that can have a sporadic or germline origin. The *RET* proto-oncogene encodes a receptor tyrosine kinase that when activated either by a point mutation or gene rearrangement can result in a constitutively active cytosolic oncoprotein (Mulligan, 2014; Chakravarty et al., 2017). Although *RET* mutations are well described in MTC (Ciampi et al., 2019), there is a paucity of information about *RET* splice variants in MTC, though one study of a MTC cell line described three splice variants, *RET* 2/4, *RET* 2/5, and *RET* 2/6 and another described functional isoforms *RET51* and *RET9*; however, neither played a role in MTC tumorigenesis (Lorenzo et al., 1995; Mule et al., 2021). Our results demonstrate that irrespective of a driving mutation, there is a high frequency of *RET* splice variants in MTC, with all 25 MTCs in this study harboring at least one *RET* splice variant *versus* only 0.3% of 3,599 non-MTCs.

The two main *RET* splice variants identified by the clinical NGS for this study were in-frame: *RET* 2/8 with skipping of exons three to seven and *RET* 2/4 with skipping of only exon 3. Although several other *RET* splice variants were identified, they were observed at a lower frequency and in conjunction with one of these two more common splice variants. The *RET* 2/8 and 2/4 splice variants were identified in the RNA-based NGS sequence; review of the accompanying DNA sequence did not reveal the causative DNA changes. The lack of detectable DNA causative changes in cases with appreciable RNA splice events has been extensively reported in the literature, particularly with the well-studied *MET* exon 14 skipping events. Numerous studies have demonstrated that DNA changes leading to these skipping events can be variable in size and position and can involve extremely large deletions bordering the intron-exon junctions (Dirlon, 2016; Poirot et al., 2017; Davies et al., 2019; Puris et al., 2020). Davies et al. demonstrated that in a cohort positive for *MET* exon 14 skipping events by RNA-based assays, DNA changes were detected only 60% of the time by DNA-based assays (Davies et al., 2019). They concluded that for accurate detection, a DNA assay should cover all regions involved in splicing, such as the branching site, polypyrimidine tract, splice acceptor and splice donor sites or an RNA-based assay should be used to directly detect the splice variants (Davies et al., 2019). Accordingly, the *RET* splice variants detected in MTC cases in this study were only identifiable by RNA-based NGS testing, with no evidence for the cause in the DNA-based sequence. The lack of DNA evidence for this change and the predominant use of DNA-based NGS may also explain why these *RET* splice variants have not been previously reported in clinical MTC cases.

Of note, the two predominant *RET* splice variants identified in our study cause skipping of exons three through 7, which encode part of the *RET* extracellular domain. This domain is distant from the intracellular tyrosine kinase domain which is altered by typical *RET* driver mutations, such as *RET* p.M918 or p. C634. Since these *RET* point mutations are so distant from our observed splicing events, we believe it is unlikely that they play any role in the observed RET splicing events.

Although the functional and clinical significance of *RET* splice variants in humans is not well characterized, two similar splice variants involving skipping of exon 3 (*RET* 2/4) and exons 3–5 (*RET* 2/6) were previously described in zebrafish, mice, and rats by Gabreski et al. (Gabreski et al., 2016) They demonstrated that *RET* 2/4 or *RET* 2/6 both translated into RET proteins with deletions in the extracellular domain that likely impacted the overall stability of the proteins. Signaling experiments demonstrated that *RET* 2/4 was phosphorylated similarly to full-length *RET*, but that *RET* 2/6 had a higher baseline autophosphorylation on one of the most important signaling residues, Tyr^1062^ (Gabreski et al., 2016). Further experiments revealed that *RET* 2/4 and 2/6 were co-expressed with the full-length *RET* transcript at several developmental time points with particularly high expression in the dorsal root ganglion in mice (Gabreski et al., 2016). Although exons 3, 4, and five are less than 50% conserved between species, these *RET* 2/4, 2/5, and 2/6 transcripts have also been identified in human kidney and substantia nigra fetal tissues (Lorenzo et al., 1995), and at very low levels (less than 1% of all *RET* transcripts) in germline (MEN2) and sporadic pheochromocytomas (Le Hir et al., 2002). The authors surmised that it is possible that *RET* RNA splicing might be dysregulated in tumor cells.

Interestingly, PTCs have been found to overexpress wild type tyrosine kinase *RET* mRNA with over expression in 70% of papillary thyroid cancers relative to expression in non-neoplastic thyroid tissue (Fluge et al., 2001; Shakiba et al., 2019). The *RET* promoter is silent in follicular cells and with wild *RET* expression but not overexpression in follicular cells and tumors derived from them. This study identified the presence of several *RET* splice variants, including *RET* 2/8 in PTC (Fluge et al., 2001). Consistent with this study, we identified *RET* 2/8 in one PTC in our study (Table 3). The authors concluded that wild-type and alternatively spliced *RET* transcripts co-exist with rearrangements in PTC and may play a role in thyroid tumorigenesis. In MTC though, high *RET* gene expression levels have not been associated with an alternative *RET* activation mechanism (Mule et al., 2021).

A couple of studies have described two *RET* protein isoforms, *RET*9 and *RET*51, that are generated by alternative splicing at the 3′ end of *RET* (C-terminus) in contrast to the 5’ end splice variants, *RET* 2/4 and *RET* 2/8, described in this study which were detected by our clinical NGS assay (Learoyd et al., 1998). The splice variants leading to the *RET*9 and *RET*51 isoforms are not covered by our clinical NGS assay. *RET*51 expression was higher in MTC than *RET*9, but both were identified in 19 patients with MTC. These *RET* isoforms displayed unique levels of auto-phosphorylation and had differential interactions with adaptor proteins and alternative splicing in intron 19 (Richardson et al., 2012; Ramone et al., 2019). These isoforms also displayed distinct subcellular localizations, trafficking properties, and downstream signaling; however, neither was demonstrated to play a role in MTC tumorigenesis (Mule et al., 2021).

The precise role of the detected alternate *RET* transcripts is unknown. It is possible that these are non-pathogenic and merely reflect altered transcriptional regulation during the carcinogenic process, perhaps reverting these cells to an earlier developmental stage, or can be attributed to the increased number of overall *RET* transcripts. Since the splice variants observed in the MTC cases have been observed during normal neural development, the consistent detection of these splice variants in MTC could reflect a non-oncogenic but cancer specific change in transcriptional regulation. However, the 4 cases in our study that lacked specific driver mutations but had multiple expressed *RET* splice variants with predicted intact kinase domains, raises the intriguing possibility that the *RET* splice variants themselves could represent the primary pathologic driver in these cases.

It has been demonstrated that while MTCs overexpress *RET* mRNA this overexpression does not play a significant role in tumorigenesis (Lian et al., 2017; Mule et al., 2021). It is possible that *RET* 2/4 and 2/8 are detected secondary to *RET* mRNA overexpression and play no significant role in tumorigenesis. However, if true, this would detract from the potential diagnostic significance for these specific splice variants.

The identification of *RET* splice variants in MTC represents a distinguishing genetic diagnostic feature of this tumor and provides an opportunity for a better understanding MTC pathogenesis. Further studies are needed to confirm the high prevalence of *RET* splice variants in MTC, to understand their cause, correlate clinically, and to investigate whether they have a silent or oncogenic role.

## Data Availability

The raw data supporting the conclusion of this article will be made available by the authors, without undue reservation.
